# Impolicy of the Apothecaries' Bill

**Published:** 1813-04

**Authors:** 


*To the Editors of the Medical and Physical Journal.*

GENTLEMEN,

HAVING retired some time from practice, but, still anxious to hear of any step towards improving the condition of the apothecary, surgeon-apothecary, and man-midwife, I accidentally got hold of an Abstract of a Bill lately brought into parliament, by the London Committee of Apothecaries, for improving their condition; but I think it liable to many objections. 1st, Are not the apothecaries, generally speaking, too poor to admit of their taking out an annual license for themselves and every dispenser of medicines cines they keep; 2dly, Imposing of 25*l.* for the stamp indenture, to go to government for every apprentice they take, is liable to great objection from the country practitioners, as it will operate very powerfully against their ever getting apprentices; 3dly, The bill bears too heavily on the chemists and druggists not to meet with powerful opposition from that quarter, whose extirpation it aims at. Now, I think, if the apothecaries were to meet the chemists and druggists on fair and reasonable terms, that some good might be effected for both parties.

I am, Gentlemen,

*London,* Your very obliged Servant,

*March* 14, 1813. SENEX.

P.S.—I think, if the present Bill passes into a law, it will add very materially to the apothecary's expenditure, without his reaping any beneficial effects from it.

